# EHMTI-0198. Importance of neurovascular conflict with the trigeminal nerve in SUNCT and SUNA

**DOI:** 10.1186/1129-2377-15-S1-C35

**Published:** 2014-09-18

**Authors:** G Lambru, P Shanahan, I Davagnanam, M Matharu

**Affiliations:** 1 Headache Group, UCL Institute of Neurology, London, UK; 2 Headache Group, The National Hospital for Neurology and Neurosurgery, London, UK; 3 Neuroradiology, The National Hospital for Neurology and Neurosurgery, London, UK; 4 Headache Group, UCL Institute of Neurology and The National Hospital for Neurology and Neurosurgery, London, UK

## Introduction

Recently, a small case series showed the presence of neurovascular conflict with the trigeminal nerve in a significant proportion of SUNCT and SUNA patients, suggesting a possible pathophysiological overlap between SUNCT, SUNA and trigeminal neuralgia (TN).

## Aims

To assess the presence of a neurovascular conflict with the trigeminal nerve in a large series of SUNCT and SUNA patients.

## Methods

Consecutive MRI examinations performed over the period from 2005 to 2013 from consecutive patients with SUNCT and SUNA syndromes were retrospectively reviewed by a consultant neuroradiologist blinded to the diagnosis and lateralisation of symptoms.

## Results

The analysis included 89 patients (SUNCT=45 patients; SUNA=44 patients). Thirty SUNCT (66.7%) and 29 SUNA patients (65.9%) had a vascular loop ipsilateral to the side of the pain. Seven SUNCT (15.6%) and three SUNA patients (6.8%) showed a vascular loop contralateral to the side of the pain, whereas eight SUNCT (17.7%) and 12 SUNA patients (27.3%) showed no vascular loops. Of the 178 trigeminal nerves considered, a vascular loop was present in 59/95 (62.1%) of the symptomatic trigeminal nerves, as opposed to 15/83 (18.1%) of the asymptomatic nerves. There was a remarkable correspondence between the site of the pain (V1, V2, V3) and the circumferential location of the site of vascular contact.

## Conclusion

The presence of neurovascular conflict with the trigeminal nerve in two-thirds of SUNCT and SUNA patients highlights their aetiological overlap with TN. The correlation between site of pain and location of the vascular contact supports the pathophysiological role of the compression of the trigeminal sensory root in SUNCT and SUNA.

No conflict of interest.

